# Films, Buckypapers and Fibers from Clay, Chitosan and Carbon Nanotubes

**DOI:** 10.3390/nano1010003

**Published:** 2011-04-06

**Authors:** Thomas M. Higgins, Holly Warren, Marc in het Panhuis

**Affiliations:** Soft Materials Group, School of Chemistry, University of Wollongong, Northfields Avenue, Wollongong, NSW 2522, Australia; E-Mails: thiggins@uow.edu.au (T.M.H.); hw982@uowmail.edu.au (H.W.)

**Keywords:** chitosan, clay, carbon nanotubes, electrical, mechanical

## Abstract

The mechanical and electrical characteristics of films, buckypapers and fiber materials from combinations of clay, carbon nanotubes (CNTs) and chitosan are described. The rheological time-dependent characteristics of clay are maintained in clay–carbon nanotube–chitosan composite dispersions. It is demonstrated that the addition of chitosan improves their mechanical characteristics, but decreases electrical conductivity by three-orders of magnitude compared to clay–CNT materials. We show that the electrical response upon exposure to humid atmosphere is influenced by clay-chitosan interactions, *i.e.*, the resistance of clay–CNT materials decreases, whereas that of clay–CNT–chitosan increases.

## 1. Introduction

Clays are excellent stabilizing and rheological agents due to their colloidal structure in water [1]. Each smectite particle is composed of thousands of platelets (thickness = 1 nm, width > 100 nm) stacked in a sandwich fashion. Hydration of the clay promotes delamination of this sandwich structure until the platelets are completely separated. This allows the weakly positive platelet edges to interact with the negatively charged platelet faces resulting in the formation of a three dimensional colloidal structure, commonly referred to as the “house of cards” [2]. The building of this structure also gives the clay time-dependent (thixotropic) rheological properties [2]. Initially, the building of the colloidal structure is rapid, leading to a sharp increase in viscosity. This increase slows down as the remaining free platelets take a longer time to find an available site in the structure. Applying a shear results in the opposite behavior, as most of the structure is disrupted leading to a decrease in viscosity [2].

The colloidal structure of clays has an ability to trap and segregate solids in suspensions, oils in emulsions, and gases in foams or mousses as well as drug delivery [1,3–5]. Recently, clays have also been used to assist with the well-known disperse-ability issue surrounding conducting fillers such as carbon nanotubes (CNTs) and carbon black (CB) in common solvents [6–12]. These studies focused on the preparation of composite materials with enhanced mechanical and/or electrical properties. For example, Tang *et al.* reported that chitosan can be reinforced through addition of clay and functionalized multi-walled carbon nanotubes (FMWNT) [7,9]. The clay–FMWNT–chitosan composite materials exhibited increased Young's modulus (125%), tensile strength (165%) and storage modulus (55%) compared to chitosan materials [7,9]. The increase in mechanical characteristics was attributed to a synergistic effect of anionic clay and anionic FMWNT on cationic chitosan through electrostatic interactions and hydrogen bonding formation. The use of electrostatic interaction in the formation of composites from oppositely charged materials is well known and generally referred to as ionic self-assembly or polyelectrolyte complexation [13].

Studies by Grunlan *et al.* investigated the electrical and mechanical characteristics of clay-epoxy composite materials with either CNTs or CB as conducting fillers [8,12]. For CNT containing composites they observed improvements in the electrical conductivity (from 0.25 mS/cm to 2 mS/cm) and lowering of percolation threshold (5-fold reduction) upon addition of clay. But the improvement in mechanical characteristics (storage modulus) was due to addition of nanotubes and not clay [8]. However, they did report synergistic effects between CB and clay resulting in improved electrical and mechanical characteristics of clay–CB–epoxy composites [12]. In contrast, they observed that clay adversely affected the mechanical and electrical behavior of clay–CB–latex materials [11]. Other research by Sue *et al.* has shown that clay–FMWNT–epoxy composite materials exhibited increased Young's modulus (40%) and tensile strength (55%) compared to epoxy materials.

In this paper, we report the mechanical and electrical characteristics of films, buckypapers and fibers prepared from combinations of clay, carbon nanotubes and chitosan. To our knowledge, these buckypapers and fibers are novel materials, which have not been reported in the literature. We show that the brittleness of clay–CNT materials can be improved through addition of chitosan, allowing the assessment of their mechanical properties. Addition of chitosan was found to decrease the electrical conductivity by up to three orders of magnitude. We also demonstrate that the addition of chitosan affects the electrical response upon hydration, providing new insights into their behavior. In addition, we show that clay–CNT–chitosan fibers can be prepared by a wet-spinning approach. The resulting fibers display higher Young's modulus, but lower conductivity values compared to the corresponding film materials.

## 2. Results and Discussion

### 2.1. Dispersing CNTs

**Figure 1.**
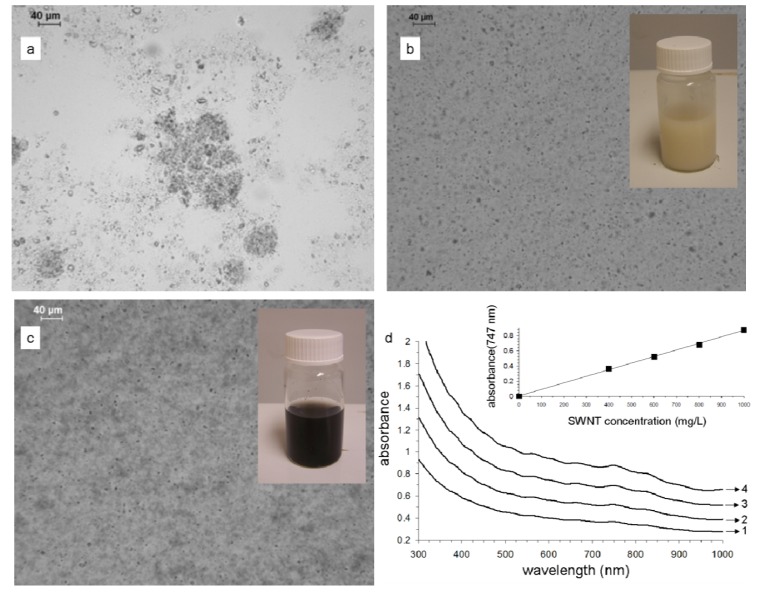
Optical microscopy images of a clay suspension: **(a)** before and **(b)** after hydration; Inset: photograph of the clay suspension after hydration; **(c)** Optical microscopy image of 800 mg/L single-walled carbon nanotubes (SWNT) dispersed in a 1.12% w/v clay suspension; Inset: photograph of the SWNT dispersion; **(d)** UV-visible absorption spectra of 400 mg/L (line 1), 600 mg/L (line 2), 800 mg/L (line 3) and 1,000 mg/L (line 4) SWNT dispersed in a 1.12% w/v clay suspension; Inset: UV-vis absorbance at 747 nm as a function of SWNT concentration in the clay suspension.

Our initial attempts to hydrate clays involving simultaneous heating (80 °C), stirring and sonicating for up to two days were unsuccessful. The resulting clay suspension was unstable and it was not possible to obtain a stable CNT dispersion. This suggested that this treatment does not fully delaminate the clay's platelet layers. Full delamination is only achieved through vigorous application of mechanical force or with the assistance of surfactants, as shown previously [14]. Therefore, all our clay suspensions were hydrated using a homogenizer. Light microscopy images (Figure 1a and b) show that the presence of aggregates is significantly reduced after hydration.

Single-walled carbon nanotubes (SWNT) were easily dispersed in these properly hydrated clay suspensions (1.12% w/v, pH = 7.9), and were stable for months (Figure 1c). Typical UV-visible spectra (Figure 1d) show broad CNT absorption features due to the presence of nanotube aggregates. The absorbance of the dispersions at 747 nm was plotted as a function of concentration (inset in Figure 1d). This particular wavelength was selected as it corresponds to the maxima of an absorption band arising from the van Hove singularities for SWNT [15,16]. Figure 1d shows that the absorption intensity increases linearly with increasing carbon nanotube concentration, indicating an excellent degree of disperse-ability (in the concentration range studied). This allowed us to determine the extinction coefficient (ε) of CNTs in the clay suspension, yielding ε= 0.864 mL mg^−1^cm^−1^.

### 2.2. Rheological Studies

**Figure 2.**
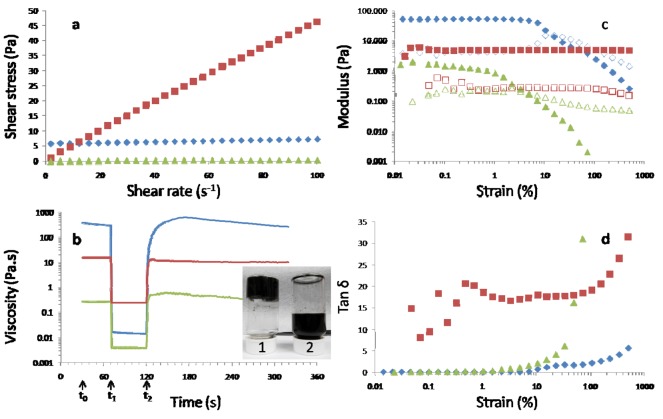
Rheological studies: **(a)** Shear stress as a function of shear rate for clay–carbon nanotube (CNT) (diamonds), clay–CNT–chitosan (triangles) and chitosan (squares); **(b)** thixotropic behavior test, viscosity as a function of time for clay–CNT (blue line), clay–CNT–chitosan (green line) and chitosan (red line). Shear rates in intervals 1, 2 and 3 are 0.01 s^−1^, 1000 s^−1^ and 0.01 s^−1^, respectively; Inset: photographs of clay–CNT suspension (1) and clay–CNT–chitosan (2) after being left undisturbed for one day; **(c)** Oscillatory amplitude sweep for clay–CNT (diamonds), clay–CNT–chitosan (triangles) and chitosan (squares). Filled and open symbols indicate storage (G′) and loss (G″) modulus, respectively; **(d)** Loss factor (tan δ = G″/G′) as a function of strain for clay–CNT (diamonds), clay–CNT–chitosan (triangles) and chitosan (squares).

Rheological studies were undertaken to examine the flow and time-dependent behavior of the clay-CNT dispersions as well as the effect of incorporating chitosan. Both types of dispersions and the chitosan solution display shear thinning behavior, *i.e.*, viscosity (η) decreases with increasing shear rate (data not shown). Combining chitosan with clay–CNT into a clay–CNT–chitosan dispersion results in a two and three orders of magnitude decrease in the apparent viscosity compared to that of the chitosan solution and clay-CNT dispersion, respectively. For example, at a shear rate of 0.01 s^−1^ the viscosity values of typical chitosan solutions, and clay–CNT (1000 mg/L) and clay–CNT–chitosan dispersions are 15.4 Pa.s, 370 Pa.s, and 0.266 Pa.s, respectively. The apparent viscosity of the ternary dispersion is lower than the oppositely charged solutions used to form the dispersion, *i.e.*, the anionic clay–CNT dispersion and the cationic chitosan solution. Figure 2a shows that the clay-CNT dispersion exhibits a yield point, *i.e.*, the sample starts to flow only when a certain amount of force is applied. This point can be determined using the Bingham model [17],

(1) $$\tau =\tau_{\text{B}}+\eta_{\text{B}}\text{s}$$

where τ_B_ and τ_B_ indicate the Bingham yield point and Bingham flow coefficient, respectively. Although, the values obtained using the Bingham model are dependent on the shear rate range it provides a good approximation for the determination of yield points. The model shows that the yield point of clay–CNT dispersion decreases by 2-orders of magnitude upon addition of chitosan (Table 1). Similar differences are observed for the Bingham flow coefficient.

These results indicate that the electrostatic interaction between the negatively charged clay and positively charged chitosan decreases the resistance against flow. Similar observations have been reported previously for the addition of other types of clay (montmorillonite) to chitosan [18]. This study showed that a decrease in the electrostatic potential of chitosan upon addition of clay was coupled with a decrease in flow resistance [18].

Thixotropic behavior testing (Figure 2b) revealed that clay–CNT materials exhibit the expected time-dependent rheology characteristics consistent with a “house of cards” structure [2]. As evident from the 20% decrease in viscosity during the reference and high-shear intervals applying a constant shear, results in disruption of this structure. During the regeneration interval, clay–CNT dispersions exhibit a rapid increase in viscosity, which is indicative of the re-building of the colloidal structure. Eventually, the viscosity will start to decrease again due to effect of applying a constant shear rate (Table 1). In contrast, chitosan does not show any of these characteristics, *i.e.*, the viscosity does not exhibit any significant time-dependent behavior in any of the three intervals. Whereas, combining chitosan with clay–CNT results in a dispersion which has retained the time-dependent characteristics of clay–CNT dispersions. The difference in the magnitude of these viscosity effects is evident from the inset in Figure 2b, *i.e.*, a binary dispersion can be easily inverted without flowing, whereas the ternary composite will still flow.

Oscillatory amplitude sweeps confirmed that the ternary (clay–CNT–chitosan) dispersion has more in common with the binary (clay–CNT) dispersion than the chitosan solution (Figure 2c–d). Both dispersions display distinctive linear viscoelastic (LVE) regions, although the length of LVE region and maximum shear stress is lower for the ternary dispersion due to the presence of chitosan (Table 1). The magnitude of the storage (G′) and loss (G″) moduli of the ternary dispersion (in the LVE region) is lower than those of the binary dispersion. This difference is also reflected in the shear modulus obtained using G* = ((G′)^2^ + (G″)^2^)^1/2^, resulting in values of 80.6 ± 1.9 Pa and 1.40 ± 0.23 Pa for the binary and ternary dispersions, respectively. The corresponding value for chitosan is 4.81 ± 0.08 Pa. The value for the clay-CNT dispersion is similar to that of typical dispersions such as lotions and creams, whereas that of chitosan and clay–CNT–chitosan is comparable to that of salad dressings [19].

Figure 2c–d shows that for the dispersions, the storage modulus (G′) is larger than the loss modulus (G″) in the LVE region, indicating that the elastic behavior dominates over the viscous behavior. In contrast, chitosan solutions exhibit the opposite trend, *i.e.*, viscous behavior is dominating (G′ < G″). Above the maximum shear strain, a cross-over from elastic to viscous behavior (tan δ > 1, Figure 2d) takes place for both dispersions indicates a disruption of the “house-of-card” structure. Furthermore, the strain at which the cross-over takes place is larger in the binary dispersion than that in the ternary dispersion. These results clearly indicate lower resistance to flow behavior due to addition of chitosan.

### 2.3. Clay–CNT Films

**Figure 3.**
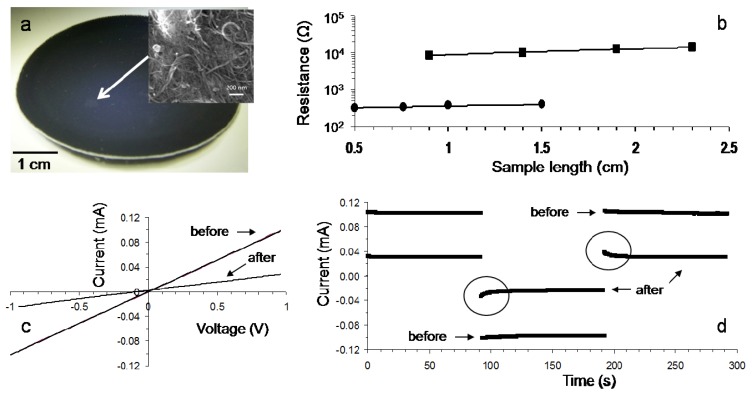
**(a)** Photograph of a typical free standing film (nanotube mass fraction = 0.067) prepared by evaporative casting of a clay–CNT composite dispersion; Inset: scanning electron microscopy image; **(b)** Resistance *versus* sample length for films prepared by evaporative casting (squares) and vacuum filtration (buckypaper, circles) under controlled ambient conditions (21 °C, 45% RH). The straight lines are fits to Equation (2); **(c)** *I*–*V* characteristics of a typical film prepared by evaporative casting before, and after exposure to humid atmosphere (21 °C, 90% RH) for 15 hours; **(d)** Current response to a square wave potential (±1 V) of a typical film prepared by evaporative casting before and after exposure to humid atmosphere (21 °C, 90% RH) for 15 hours. The circled areas highlight the non-linear behavior of the current response of the film after exposure to humid atmosphere.

Free-standing clay–CNT films (Figure 1a) were prepared by evaporative casting of clay–CNT dispersions. The current–voltage (*I*–*V*) characteristics were investigated under controlled ambient conditions (21 °C, 45% relative humidity, RH). All films exhibited linear *I*–*V* characteristics, which indicate Ohmic behavior. The conductivity (*σ*) can then be evaluated by making resistance measurements as a function of sample length (*l*) [20]. The total resistance was found to scale linearly according to:

(2) $$R_{T}=\frac{1}{\sigma A_{C}}l+R_{C}$$

where *A_c_* is the film's cross-sectional area. The straight line fit for a typical film with nanotube mass fraction 0.067 is shown in Figure 3b. The slope is used to calculate the so-called two-probe dc conductivity, yielding 0.14 ± 0.04 S/cm under controlled ambient conditions.

Previously, we have demonstrated that (dried) CNT composite materials prepared using water soluble dispersants change their electrical behavior upon hydration. For example, exposure to a humid atmosphere resulted in an increase in electrical resistance for water soluble polyaniline and polypeptide-CNT composite materials [21,22], while gellan gum–CNT composite materials decrease their resistance [20,23,24]. It was demonstrated that resistance decreased due to an increased cation mobility upon exposure to humid atmosphere [20].

Figure 3c shows that exposing our clay–CNT film to humid atmosphere for 15 hours results in a decrease in the current compared to that observed under ambient conditions. This decrease in current corresponds to an increase in electrical resistance, from 9.7 ± 2.0 kΩ (R_B_, before exposure) to 36 ± 4 kΩ(R_A_, after exposure). Exposure to the humid atmosphere results in hydration of the clay–CNT film, *i.e.*, osmotic forces drive water in between the smectite platelet galleries. This leads to a swelling-induced disruption of conductive pathways resulting in an increase in resistance.

Figure 3d shows that the current response to a square wave potential is different before and after exposure to humid conditions. Under ambient conditions (before exposure) the magnitude of the current response to a square wave potential is linear, while after exposure to humid atmosphere the current displays non-linear behavior.

This behavior can be explained through the mobility and charge collection of the counter-ions. Under an applied positive potential the counter-ion (cations) will migrate towards the negative electrode (1) leading to a buildup of positive charge. Upon reversal of the potential, the cations will be repelled from the now positive electrode 1 causing a non-linear current flow due to migration of the ionic charge carriers (indicated in the circled area in Figure 3d). The cations migrate towards the negative electrode (2) leading to a charge collection at this electrode. This effect manifests itself as the non-linear current response, until all mobile ions have migrated and the current becomes linear again.

Thus, the resistance of our composite material consists of an electrical contribution from electron transport through the carbon nanotube network and an ionic contribution due to the cations. The latter is small or negligible under ambient conditions. Under humid conditions we would expect a decrease in resistance due to an increased ionic contribution, similar to that observed in our previous work on composites consisting of the anionic polysaccharide gellan gum and SWNT [20,24]. However, the swelling-induced disruption of conductive pathways results in a more significant reduction in the electrical contribution (−70%, estimated from Figure 3d). As such the resistance of a hydrated film is higher compared to that of a dry film.

Clay–CNT dispersions were used to fabricate buckypapers via vacuum filtration. The two-probe dc conductivity of a typical buckypaper yielded 0.9 ± 0.2 S/cm under controlled ambient conditions (Figure 3b). As expected, the buckypaper conductivity is higher compared to the conductivity (0.14 ± 0.04 S/cm) of the evaporative cast film. Exposure of buckypapers to humid atmosphere resulted in a swelling-induced decrease in the current (increase in resistance), but we did not observe any non-linear current behavior in response to a square wave potential. This indicates that most of the counter-ions were removed during the washing procedure in the buckypaper preparation method.

### 2.4. Clay–CNT–Chitosan Films

The clay–CNT films produced by evaporative casting and vacuum filtration were too brittle to allow a detailed analysis of their mechanical properties, *i.e.*, the films could not be subjected to any significant strain without breaking. Polyelectrolyte complexation of the negatively charged, hydrated clay platelets with the positively charged biopolymer chitosan was utilized to improve the mechanical robustness of these materials, *i.e.*, the materials could be subjected to strain.

**Figure 4.**
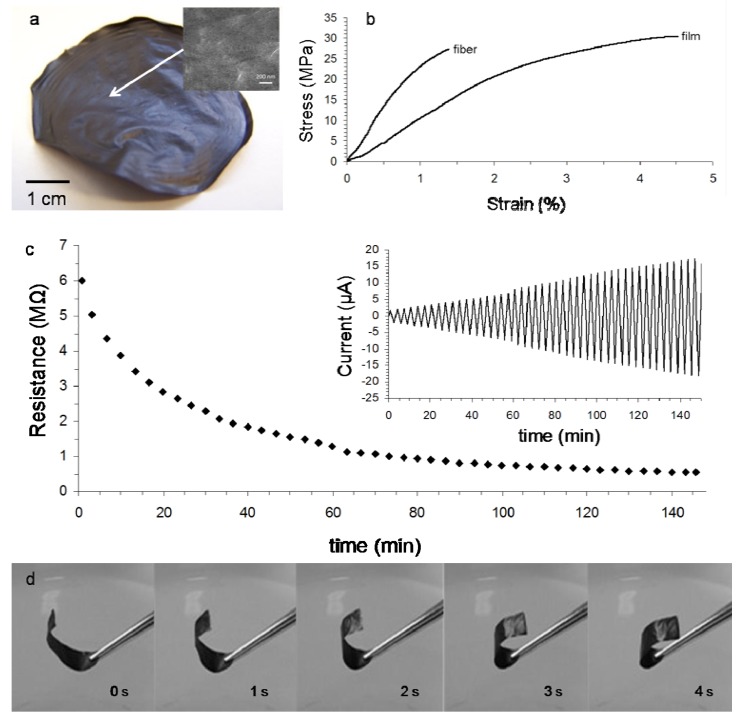
**(a)** Photograph of a typical free standing film (nanotube mass fraction = 0.028) prepared by evaporative casting of a clay-CNT-chitosan composite dispersion; Inset: scanning electron microscopy image; **(b)** Stress–strain curves for typical clay–CNT–chitosan fibers and typical free standing film (nanotube mass fraction = 0.028) prepared by evaporative casting of a clay–CNT–chitosan composite dispersion; **(c)** Typical film resistance as a function of time during exposure to humid atmosphere (21 °C, 90% RH) for 150 minutes; Inset: current response of the film to a triangular wave potential of during exposure to humid atmosphere; **(d)** Video images showing the actuatory response of a typical film when exposed to water vapor. Numbers indicate the time lapsed with respect to the first image.

Free-standing ternary clay–CNT–chitosan composite films (Figure 4a) were prepared by evaporative casting of clay–CNT–chitosan dispersions with CNT mass fraction of 0.028. The resulting materials were more mechanically robust compared to clay–CNT films, allowing for an assessment of their mechanical properties (see Figure 4b). Combining clay–CNT with chitosan results in an improvement in Young's modulus (E), coupled with a decrease in tensile strength and strain at break values compared to chitosan (Table 2). More significant increases in E as well as an increase in TS have been observed for composites prepared using functionalized multi-walled carbon nanotubes (FMWNT, see also Table 2) [7,9]. This larger increase can be attributed to the presence of carboxy and hydroxyl functional groups on the nanotube surface, which facilitates an improved interfacial adhesion between clay and chitosan through electrostatic interactions and hydrogen bonding, compared to the non-functionalized SWNT used in our composites. Larger increases in modulus were also observed for composites prepared using other matrix materials (epoxy and latex) in combination with carbon black and FMWNT (Table 2) [8,10–12].

The increased robustness of the ternary (clay–CNT–chitosan) composite materials is coupled with a decrease in conductivity by 3-orders of magnitude (from 0.14 S/cm to 1.0 × 10^−4^ S/cm) compared to the binary (clay–CNT) composites, see Table 2. These observations suggest that chitosan may act as “glue” or “binder” between the clay–CNT domains thereby improving the mechanical properties, as suggested previously [25]. However, the significant reduction in conductivity suggests that the number of electrical (CNT–CNT) pathways has decreased and the number of ionic-electrical pathways has increased compared to clay-SWNT films, *i.e.*, pathways dominated by chitosan and clay–chitosan. This is evident from the difference in surface morphology between the two types of films. The CNT pathways are clearly visible in the clay–CNT film (Figure 3a), but almost entirely covered by the biopolymer in the clay–CNT–chitosan film (Figure 4a). We were unable to compare our conductivity values with that of the other clay–CNT–chitosan materials shown in Table 2, due to lack of available data (at least to our knowledge). However, our conductivity value is in the same order of magnitude as clay–CNT–epoxy materials, with higher values (8.6 mS/cm) reported for carbon black (CB) containing materials (Table 2).

Under ambient conditions (in the absence of water vapor), chitosan and clay act as tunneling barriers in these junctions thereby blocking transport. We have already seen that exposure to humid atmosphere of clay–CNT materials results in an additional contribution to the current. As chitosan is a cationic polyelectrolyte, exposure to humid atmosphere increases the counter-ion mobility allowing these anionic charge carriers to transport the current along the polymer component of the chitosan-dominated junctions. This may enable transport through these pathways leading to an additional contribution to the current.

Despite the increase in current (as a result of increased ion-mobility), the resistance of the clay–CNT films increased upon exposure to humid atmosphere due to a swelling effect. Figure 4c shows that the clay–CNT–chitosan films exhibit different behavior. The current magnitude increases with increasing time of exposure to humid atmosphere. After 140 min of exposure the resistance has decreased by one order of magnitude from R_B_ = 2.8 ± 0.6 MΩ to R_A_ = 0.27 ± 0.08 MΩ (see also Table 2). It is likely that interactions between the oppositely charged clay and chitosan materials limits expansion (swelling) of the clay. As such swelling-induced disruption of conductive pathways (resulting in an increase in resistance) is not significant in these composites. The decrease in resistance can then be attributed to enhanced ion-mobility of the clay and chitosan counter-ions.

These ternary composite materials showed another interesting and somewhat unexpected response to humidity. Exposing one face of the film to a higher humidity than the other face, results in rapid curling (Figure 4c). This response was found to be reversible, *i.e.*, the film uncurled upon removal of the humidity gradient. This may suggests that water is adsorbed into the inter-layer spacing on only one side of the film; expansion of that side relative to the other (dryer) side results in the curling actuator response. The actuator response (the level of reversible curling) was better for dry films compared to hydrated films. The latter do not exhibit the same degree of actuation as transport of water in and out of the film becomes more uniform and with it, the amount of expansion.

### 2.5. Clay–CNT–Chitosan Fibers

In our previous work we prepared fibers by facilitating polyelectrolyte complexation through injection of a SWNT-biopolymer dispersion into a coagulation bath containing a biopolymer of opposite charge [23]. Initial attempts to produce fibers via this approach, *i.e.*, injection of a clay–CNT dispersion into a chitosan coagulation bath, were unsuccessful. The resulting fibers were not mechanically robust enough to be recovered after passing through the coagulation bath. We suspect that this may be a result of the high yield strength (5.87 Pa) and apparent viscosity (370 Pa.s at 0.01 s^−1^) of the clay–CNT dispersion which may inhibit the diffusion of chitosan and subsequent coagulation of chitosan with the clay platelets. In other words, during the continuous spinning approach the clay–CNT dispersion is passed too quickly through the chitosan coagulation bath to facility polyelectrolyte complexation.

We devised an alternative spinning method whereby the chitosan coagulation bath is replaced by a long coagulation channel into which a stream of a clay–CNT dispersion is injected, which remains in the channel for three hours. This is followed by removing the fiber from the channel to a supporting frame and drying under controlled ambient conditions. We refer to this modification of the continuous spinning approach as “stop-and-go wet spinning”. During the “stop stage”, the additional three hours in the coagulation channel, chitosan diffuses into the clay thereby facilitating the polyelectrolyte complexation. The gradual inclusion of the chitosan between the smectite platelets, causes a reduction in the thickness of the charged double layer responsible for face-face electrostatic repulsion of adjacent clays platelets. The observed shrinkage of the fibers is in support of this suggestion.

**Figure 5.**
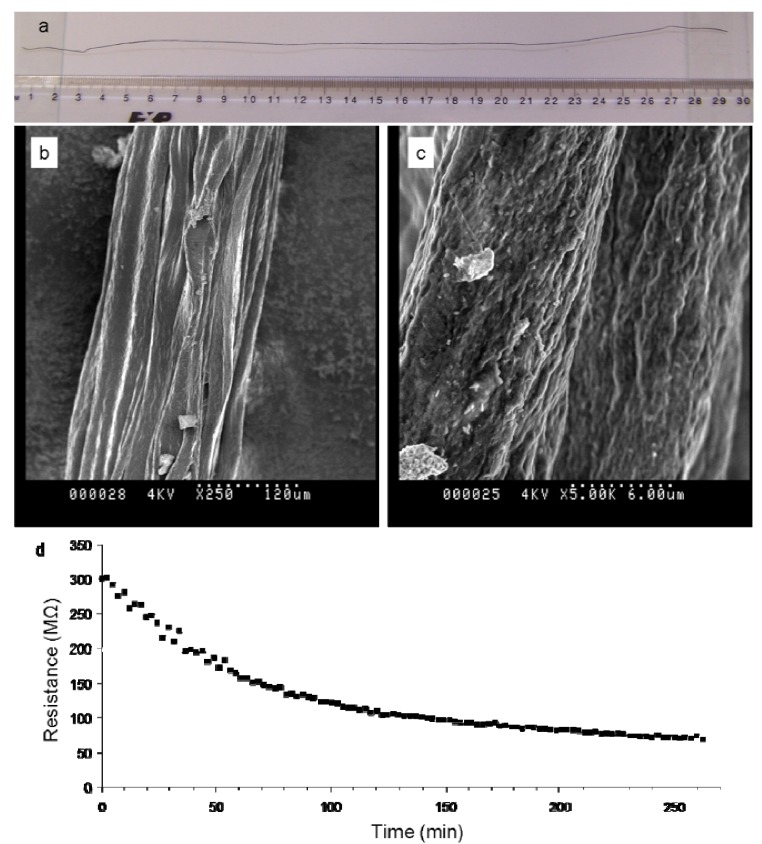
**(a)** Photograph of a typical clay–CNT–chitosan fiber; **(b, c)** Scanning electron microscopy images of the fiber's surface morphology; **(d)** Typical fiber resistance as a function of time during exposure to humid atmosphere (21 °C, 90% RH) for 270 minutes.

The stop-and-go spinning method allowed us to easily spin clay–CNT–chitosan fibers (Figure 5a). These fibers (diameter 210 ± 40 μm) showed an interesting surface morphology as evident from the scanning electron microscopy micrographs (Figures 5b and 5c). These ternary composite materials appear to be composed of numerous smaller fibers (diameter 23 ± 9 μm), producing a yarn like appearance. Similar surface features have been reported for other types of polyelectrolyte complexed fibers using gellan gum and chitosan solutions [26].

Figure 4b and Table 2 clearly show that a typical ternary composite fiber exhibits significantly higher E, similar TS and lower strain at break values compared to a typical ternary composite film. The electrical resistance of typical dry fibers (R_B_ = 300 ± 14 MΩ) is two order of magnitude higher compared to typical dry films of similar length, but due to the difference in the cross-sectional area of fiber and film samples the difference in conductivity is only 1 order of magnitude (Table 2).

The fiber's electrical response to humid atmosphere is similar to that observed for clay–CNT–chitosan films. After 250 min of exposure the resistance has decreased by almost one order of magnitude from R_B_ = 300 ± 14 MΩ to R_A_ = 68 ± 4 MΩ. Swelling of the fiber in response to exposure to humid atmosphere was apparent through elongation of the fiber (+20%) within its constrained position in the environmental chamber. This swelling behavior was found to be reversible. Similar to the ternary film composites we do not consider the swelling-induced disruption of conductive pathways (resulting in an increase in resistance) to be significant in the fibers. As such the decrease in resistance is attributed to enhanced ion-mobility of the clay and chitosan counter-ions.

## 3. Experimental Section

### 3.1. Materials

Purified SWNTs produced by the HiPco process by catalytic chemical vapor deposition were obtained from Unidym (Lot P0341). Sodium smectite clay (cationic exchange capacity 80–100 meq/100 g, lot 6D-904) was a gift from R.T. Vanderbilt. Chitosan (high molecular weight, 75.6% degree of deacetylation, product number 419419, lot number 10305DD) was obtained from Sigma Aldrich. All materials were used as received. Clay dispersions (2.0% w/v) were prepared by slowly adding 12 g of as-received clay powder to 600 mL Milli-Q water (∼80 °C), and homogenized at ∼10,000 rpm (Tokushu Kika Homo Mixer) for 40 min at 80 °C. The clay dispersions were centrifuged (Heraeus Labofuge 300) for 5 min at 2,000 rpm prior to usage resulting in a clay concentration of 1.12% w/v. Homogeneous SWNT dispersions were prepared by the probe sonication process in a water bath (Digital Branson Sonifier) utilizing a power output of 120 W for 24 min and 40 W for 3 min in pulse mode (0.5 s on/off), respectively. Different amounts of SWNT (0.040% w/v, 0.060% w/v, 0.080% w/v, 0.10% w/v) were dispersed in a clay dispersion. Chitosan solutions (1.0% w/v) were prepared by dissolving chitosan powder in acetic acid (2.0% w/v) under continuous stirring at 40 °C. Clay–SWNT–chitosan dispersions were prepared by combining equivalent amounts of clay–SWNT dispersions (SWNT concentration = 0.060% w/v) with chitosan solutions, followed by sonication at 40W for 3 min in pulse mode (0.5 s on/off).

### 3.2. Film Preparation

Free-standing films were prepared by evaporative casting of clay-CNT, and clay-CNT-chitosan composite dispersions onto plastic substrates. Five mL of dispersion was injected into the base of a cylindrical plastic container (diameter ∼5.5 cm) and dried under controlled ambient conditions, 21 °C, 45% relative humidity (RH) for ∼36 hours. The films were then peeled off the substrate to yield uniform free-standing films. Buckypapers were prepared by vacuum filtration of clay–CNT dispersions. The clay–CNT dispersion was prepared by diluting 30 mL of a dispersion (0.10% w/v SWNT, 1.12% w/v clay) with 70 mL Milli-Q water and subsequently suction filtered at 30–50 mbar. Once the dispersion had been filtered, the resulting buckypaper was washed with 250 mL Milli-Q water followed by methanol (99.8%) whilst still in the filtration unit.

### 3.3. Fiber Spinning

Fibers were prepared using a custom-made fiber preparation system, consisting of a coagulation channel containing coagulant solution (1.0% w/v chitosan) confined to linear motion by a channel path guide, a syringe pump for injecting spinning solution into the coagulation channel, and a constant velocity motor-driven spool assembly to pull the coagulation channel through the path guide, away from the syringe. A 5 mL syringe with a detachable needle (diameter = 0.60 mm) controlled by a syringe pump (KDS Scientific-100) was used to deliver the clay–CNT spinning dispersion (CNT concentration = 0.060% w/v) at 249 mL/min to the coagulation channel, while simultaneously pulling the coagulation channel away from the needle at 2 cm/s. The freshly formed fiber was allowed to remain in the coagulation channel for 3 hours. The resulting composite fibers were washed and dried in air under tension.

### 3.4. Characterization

The absorption behavior of clay-CNT dispersions was obtained using a Cary 500 UV-Vis-NIR and quartz cuvette (1 cm pathlength). Rheological testing was conducted using an Anton Paar–Physica MCR 301 parallel plate rheometer working with a 50 mm head at 21 °C. CNT dispersions and chitosan solutions were analyzed using flow curves (viscosity and shear stress *vs.* shear rate), thixotropy tests and oscillatory amplitude sweeps. The thixotropy behavior was carried out using a shear rate profile with three intervals as a step function, *i.e.*, shear rate = 0.01 s^−1^ for 40 s, shear rate = 1000 s^−1^ for 30 s and shear rate = 0.01 s^−1^, for 180 s. These three intervals are hereafter referred to as: “reference interval”, “high-shear interval”, and “regeneration interval”, respectively. Oscillatory amplitude experiments were obtained at constant oscillation frequency of 1.6 Hz.

For conductivity measurements, films (cut into strips of 0.5 cm × 3.0 cm) and fibers (cut to 3.0 cm in length) were contacted with conducting silver paint. Current (*I*)–voltage (*V*) characteristics were obtained by measuring current using a digital multimeter (Agilent 34410A) under a cycling potential applied by a waveform generator (Agilent 33220A). *I*–*V* measurements were conducted under controlled ambient conditions in air (21 °C, 45% RH) as a function of film length, by repeatedly cutting the end off the strip, contacting with silver and re-measuring the *I*–*V* characteristics. Film thicknesses and fiber diameters (*d_fiber_*) were determined using a Mitutoyo digital micrometer and a Leica macroscope (Z16 APO), respectively.

The electrical responses of film and fiber samples to a humid environment were determined using an in-house designed sealed environmental chamber. *I*–*V* characteristics were conducted under controlled ambient conditions (21 °C, 45% RH) as well as during and after exposure to a humid atmosphere (21 °C, 90% RH for ∼15 hours) through measurement of the current response to applied sawtooth and square wave potentials, cycling at 5 mHz. The exposure area of films and fibers is 2.0 cm^2^ and π*d_fiber_* × 1 cm^2^, respectively.

The mechanical properties were determined using a Instron 5566 at a strain rate of 0.1 mm min^−1^. Film samples were cut into strips of 5 × 30 mm^2^ and their thicknesses were measured using the digital micrometer. Fiber samples were mounted on aperture cards (1 cm length window) with commercial superglue and allowed to air dry. Stress is calculated from the load (in Newtons) per cross-sectional area. The cross-sectional area *A* of fibers is estimated using *A* = *¼π(d_fiber_)^2^*. Strain is obtained from the ratio of the increase in sample length (*Δl*) and the initial sample length (*l_0_* = 1.0 cm) during a tensile test. Young's modulus and tensile strength values are calculated from the slope of the linear part of the stress-strain curve and the maximum stress, respectively.

Scanning electron microscopy (SEM) was carried out on a Hitachi S-900 field emission SEM through the Australian Microscopy and Microanalysis Research Facilities at the University of New South Wales (Sydney, Australia).

## 4. Conclusions

In this paper, the production of conducting films, buckypapers and fibers from combinations of clay, SWNT and chitosan is reported. Rheological studies showed that although interactions between clay and chitosan decrease the magnitude of apparent viscosity, the clay's time-dependent characteristics are maintained. The conductivity of films and buckypapers prepared from clay–SWNT dispersion is 0.14 ± 0.04 S/cm and 0.9 ± 0.2 S/cm, respectively. Hydration through exposure to humid atmosphere resulted in enhanced ion mobility (decrease in resistance) as well as swelling (increase in resistance). The increased resistance indicated that the effect of swelling (resulting in disruption of conducting pathway) was larger than the ion contribution. Clay–SWNT materials were found to be too brittle to allow assessment of their mechanical properties. The addition of chitosan increased their mechanical robustness, but resulted in a decrease of more than 3-orders of magnitude in conductivity (from 140 mS/cm to 0.8 mS/cm) compared to clay–SWNT materials. In contrast, the resistance of clay–SWNT–chitosan films decreases by an order of magnitude upon exposure to a humid atmosphere for two hours. This indicated that due to the presence of chitosan the effect of swelling on the resistance is not significant in these composites. Rather, the decrease in resistance can be attributed to ion mobility.

We also prepared clay–SWNT–chitosan fibers using a wet-spinning approach. Polyelectrolyte complexation was facilitated by injecting an anionic clay–SWNT dispersion into a coagulation bath containing the cationic biopolymer chitosan. The fiber materials exhibited higher Young's modulus (2.3 GPa), but lower tensile strength (23 MPa), strain at break (1.2%) and conductivity (0.10 mS/cm) values compared to corresponding clay-SWNT-chitosan films. The fibers displayed similar electrical response upon hydration compared to film materials, *i.e.*, an order of magnitude decrease in electrical resistance. This work contributes to the development of clay-based film and fiber materials.

## Figures and Tables

**Table 1.** Summary of rheology analysis of typical clay–CNT dispersion, clay–CNT–chitosan dispersion and chitosan solution. Bingham yield point (τ_B_) and Bingham flow coefficient (η_B_) values were obtained using the Bingham model, over shear rate range 1–100 s^−1^ (Figure 2a). η_ref_ and η_high_ are the apparent viscosity values at the end of the reference interval (shear rate = 0.01 s^−1^) and high-shear (shear rate = 1000 s^−1^) intervals during the thixotropic behavior test (Figure 2b). η_30_, η_60_, η_120_, and η_180_ indicate the apparent viscosity during the regeneration interval (shear rate = 0.01 s^−1^) at 30, 60, 120 and 180 s, respectively. Values in square brackets indicate percentage values. τ_max_ and γ_max_, refer to the maximum shear stress and shear strain of the linear viscoelastic region observed in the amplitude sweep profiles (Figure 2c).

**Sample**	**τ_B_ (Pa)**	**η_B_ (Pa.s)**	**η_ref_ (Pa.s)**	**η_high_ (Pa.s)**	**η_30_ (Pa.s)**	**η_60_ (Pa.s)**	**η_120_ (Pa.s)**	**η_180_ (Pa.s)**	**τ_max_ (Pa)**	**γ_max_ (%)**
Clay–CNT	5.87 ± 0.02	0.0153 ± 0.0003	287 ± 34	0.0142 ± 0.0001	433 ± 24	601 ± 5	405 ± 7	297 ± 5	2.0 ± 0.5	4.0 ± 1.1
Clay–CNT–Chitosan	0.017 ± 0.001	0.00429 ± 0.00001	0.265 ± 0.008	0.0040 ± 0.0002	0.596 ± 0.006	0.53 ± 0.03	0.384 ± 0.008	0.23 ± 0.03	(1.3 ± 0.5) × 10^−3^	0.10 ± 0.02
Chitosan	1.19 ± 0.14	0.458 ± 0.002	15.4 ± 0.1	0.24 ± 0.1	11.1 ± 0.1	10.7 ± 0.1	10.0 ± 0.1	10.1 ± 0.2	n.a.	n.a.

**Table 2.** Summary of Young's/storage modulus (Modulus), tensile strength (TS), strain at break (γ), electrical conductivity (σ), ratio of resistance after (R_A_) and before (R_B_) exposure to humid atmosphere for the different materials prepared by combining sample 1 with sample 2. For example, a combination of clay-SWNT and chitosan indicates a clay–SWNT–chitosan material. Differences between the sample materials are indicated by numbers, e.g., clay1 is montmorillonite clay, while clay2 is a model clay consisting of ZrP platelets. FMWMT1–2, chitosan1–2 and CB indicate different types of chitosan, different types of functionalized multi-walled carbon nanotubes and carbon black, respectively, see tabulated references for complete details. Storage modulus values indicated by * (errors not stated in source).

**Sample 1**	**Sample 2**	**Modulus (GPa)**	**TS (MPa)**	**γ (%)**	**σ (mS/cm)**	**R_A_/R_B_**	**Source**
Chitosan	-	1.2 ± 0.2	39 ± 5	10 ± 2	0	-	This work-film
Clay–SWNT	-	-	-	-	140 ± 40	3.7 ± 1.2	This work-film
Clay–SWNT	Chitosan	1.4 ± 0.2	25 ± 6	5.8 ± 1.3	0.80 ± 0.20	0.10 ± 0.05	This work-film
Clay–SWNT	Chitosan	2.3 ± 0.2	23 ± 4	1.2 ± 0.2	0.10 ± 0.01	0.23 ± 0.02	This work-fiber
Chitosan1	-	1.40 ± 0.05	43 ± 2	12 ± 3	-	-	7-film
Chitosan1–Clay1	FMWNT1	3.14 ± 0.03	114 ± 5	7 ± 2	-	-	7-film
Chitosan2	-	3.771 *	-	-	-	-	9-film
Chitosan2–Clay1	FMWNT2	5.889 *	-	-	-	-	9-film
Epoxy1	-	3.06 *	-	-	0	-	8-film
Clay1–SWNT	Epoxy1	3.73 *	-	-	2	-	8-film
Clay1–CB	Epoxy1	4.31 *	-	-	0.016 ± 0.002	-	12-film
Latex	-	2.3 *	-	-	0	-	11-film
Latex–CB	Clay1	2.54 *	-	-	8.6	-	11-film
Epoxy2	-	3.04 ± 0.04	75 ± 4	3.7 ± 0.1	-	-	10-film
Clay2-FMWNT2	Epoxy2	4.27 ± 0.07	116 ± 6	4.3 ± 0.4	-	-	10-film
